# Out with AI, in with the psychiatrist: a preference for human-derived clinical decision support in depression care

**DOI:** 10.1038/s41398-023-02509-z

**Published:** 2023-06-16

**Authors:** Marta M. Maslej, Stefan Kloiber, Marzyeh Ghassemi, Joanna Yu, Sean L. Hill

**Affiliations:** 1 Krembil Centre for Neuroinformatics, Centre for Addiction and Mental Health, Toronto, ON Canada; 2 Campbell Family Mental Health Research Institute, Centre for Addiction and Mental Health, Toronto, ON Canada; 3 Department of Psychiatry, University of Toronto, Toronto, ON Canada; 4 Institute for Medical Engineering & Science, Massachusetts Institute of Technology, Cambridge, MA USA; 5 Department of Electrical Engineering and Computer Science, Massachusetts Institute of Technology, Cambridge, MA USA; 6 Vector Institute for Artificial Intelligence, Toronto, ON Canada

**Keywords:** Health sciences, Depression

## Abstract

Advancements in artificial intelligence (AI) are enabling the development of clinical support tools (CSTs) in psychiatry to facilitate the review of patient data and inform clinical care. To promote their successful integration and prevent over-reliance, it is important to understand how psychiatrists will respond to information provided by AI-based CSTs, particularly if it is incorrect. We conducted an experiment to examine psychiatrists’ perceptions of AI-based CSTs for treating major depressive disorder (MDD) and to determine whether perceptions interacted with the quality of CST information. Eighty-three psychiatrists read clinical notes about a hypothetical patient with MDD and reviewed two CSTs embedded within a single dashboard: the note’s summary and a treatment recommendation. Psychiatrists were randomised to believe the source of CSTs was either AI or another psychiatrist, and across four notes, CSTs provided either correct or incorrect information. Psychiatrists rated the CSTs on various attributes. Ratings for note summaries were less favourable when psychiatrists believed the notes were generated with AI as compared to another psychiatrist, regardless of whether the notes provided correct or incorrect information. A smaller preference for psychiatrist-generated information emerged in ratings of attributes that reflected the summary’s accuracy or its inclusion of important information from the full clinical note. Ratings for treatment recommendations were also less favourable when their perceived source was AI, but only when recommendations were correct. There was little evidence that clinical expertise or familiarity with AI impacted results. These findings suggest that psychiatrists prefer human-derived CSTs. This preference was less pronounced for ratings that may have prompted a deeper review of CST information (i.e. a comparison with the full clinical note to evaluate the summary’s accuracy or completeness, assessing an incorrect treatment recommendation), suggesting a role of heuristics. Future work should explore other contributing factors and downstream implications for integrating AI into psychiatric care.

## Introduction

Advances in artificial intelligence (AI) are facilitating the development of clinical support tools (CSTs). AI-based CSTs can assist with reviewing patient information, informing diagnosis, and selecting optimal treatments [1]. CSTs powered by deep learning algorithms are emerging in areas such as oncology, diabetes, and cardiology [2]. Although only a handful of CSTs have been validated in clinical settings, virtually all clinicians are anticipated to interact with some form of AI technology in the future [2]. CSTs are not novel concepts; computer-derived graphical summaries of patient data have been described decades ago [3]. The integration of AI into these systems is only recently being explored. According to validation studies, however, the success of AI-based CSTs in experimental settings often does not hold up in the real world. There are examples of tools providing potentially harmful recommendations for treating patients with cancer or pneumonia [4]. This limited performance in clinical settings makes it critical to understand how clinicians will interact with AI-based information, particularly when it is incorrect. Furthermore, improving the accuracy of AI does not always translate to enhanced clinical performance [1], suggesting that contextual factors, like perceptions about AI, may shape interactions. To investigate this topic, we conducted an experiment to examine psychiatrists’ perceptions of AI-based CSTs and how perceptions interact with the quality of CST information.

### Background

In psychiatry, CSTs are being developed to assess and monitor symptoms of mental illness and provide targeted or personalised care. A major challenge in major depressive disorder (MDD) is related to the selection of treatment. Various pharmacological and psychosocial treatments for MDD are available, but it is unclear which treatments work best for which patients. Researchers are training machine learning models on clinical data to predict treatment response in MDD, with the aim of developing AI-based CSTs that can match patients with optimal treatments [5, 6]. Other CSTs for psychiatric care may emerge from advances in text summarisation. Clinical notes from electronic health records are integral for documenting and guiding patient care, but their review is often limited by time constraints and attentional demands [7]. AI-based methods are being developed to process and summarise clinical notes [8]. In particular, transformer-based language models are contributing to benchmark performance in extracting relevant information from clinical text [9]. Future applications of this technology may involve CSTs capable of generating AI-based abstractive summaries of clinical notes.

Given efforts to integrate AI into clinical decision support, there is a need for empirical research into how the users of this technology, like psychiatrists, will interact with it. AI applications in healthcare are anticipated to be collaborative, with clinicians consulting AI to inform assessment or care [10]. Thus, successful AI integration depends on a willingness on the part of clinicians to accept and interact with this technology. Although low user acceptance of AI-based CSTs in MDD has been attributed to a failure to consider user needs and expectations in CST design [1], perceptions of AI may also play a role. These perceptions can be diverse, with preferences observed for both human and AI-based decision support [11, 12]. In psychiatry, it has been argued that AI is unlikely to outperform human prognostication, due to the heterogenous biological underpinnings of most disorders and the phenotypic nature of assessment [13]. These challenges may contribute to scepticism among psychiatrists about the current utility of AI for treating MDD. On the other hand, qualitative work suggests that clinicians are receptive and willing to use AI-based tools [14]. One study evaluated how psychiatrists and residents engaged with an AI-based CST for treatment selection in MDD within a simulated patient encounter. In qualitative interviews, participants reported they were willing to use the CST in their clinical practice, even for complex or treatment-resistant patients. Importantly, most participants trusted the AI’s recommendations and found them to be clinically useful [15]. Psychiatrists may therefore have favourable perceptions of AI-based CSTs, but the study did not examine perceptions of human-derived information as a comparison. Furthermore, the CST’s recommendations were based on standardised guidelines for treating MDD [15], making it unclear how the quality of the recommendation would impact perceptions.

There is concern that clinicians may over-rely on AI-based recommendations, even if they are incorrect. According to one qualitative study, perceptions of a deployed AI-based CST for assessing the risk of sepsis were generally positive, but nurses and physicians expressed concerns that over time, clinicians would rely less on their judgement and default to the tool’s recommendation [16]. Other studies provide experimental support for these concerns, additionally finding that clinical expertise and familiarity with AI may play a role. In one experiment [17], researchers examined responses to diagnostic advice, based on whether physicians believed the advice was generated by human experts (i.e. radiologists) or AI systems. All advice was generated by radiologists, but in two cases, it was incorrect. Physicians with more expertise in the diagnostic task (i.e. radiologists) tended to agree less with incorrect advice provided by the AI. Nevertheless, almost a third of radiologists always followed incorrect advice, regardless of whether it was AI- or human-derived [17]. A negative impact of incorrect advice on treatment decisions also emerged in a study of psychiatrists [18]. In this experiment, psychiatrists were asked to select among antidepressant treatments for hypothetical patients with MDD with AI-based recommendations that were either correct (top-scoring antidepressants rated by pharmacologists) or incorrect (lowest-scoring antidepressants). Although psychiatrists rated the incorrect recommendations to be less useful, they made less accurate treatment decisions when presented with incorrect recommendations [18]. Interestingly, psychiatrists who reported being less familiar with AI methods were more likely to follow the AI-based recommendation [18].

Paired with concerns emerging from qualitative research [16], findings from these experiments suggest that when AI-based CSTs provide incorrect information, they may have unintended, negative impacts on clinical care. Prior to deployment, it may be necessary to provide information about their limitations, as well as guidance on how they should be used. At the same time, expertise (either clinical or in AI) may moderate these effects, which would provide some preliminary evidence into the types of populations that may be particularly susceptible (e.g. residents or physicians without specialised expertise in the clinical task) or the types of interventions that may be required (e.g. training to increase familiarity with AI).

### Aims

We conducted an experiment to evaluate how psychiatrists perceive the information provided by two CSTs for treating individuals with MDD: a clinical note summary and treatment recommendation. Our primary aim was to investigate perceptions of AI-based CST information, and how these perceptions interact with information quality. To address this aim, we compared ratings of CSTs based on their perceived source (as AI or another psychiatrist), and when they provided correct and incorrect information. As a secondary aim, we examined whether CST information quality interacted with clinical experience and familiarity with AI. Based on favourable impressions of AI-based CSTs observed in prior work [16], we expected that psychiatrists might rate information provided by CSTs more favourably if they believed it was generated with AI, as compared to another psychiatrist, regardless of its quality. However, we predicted that psychiatrists would rate low-quality information more favourably if they had less clinical experience [17]. We also anticipated that psychiatrists who were less familiar with AI might rate low-quality information derived with AI more favourably, as compared to psychiatrists more familiar with AI [18].

## Methods

### Participants

Psychiatrists were primarily recruited from a large, Canadian psychiatric hospital, with a small subset recruited from four other institutions in the same province. All institutions provide a range of clinical services for patients with mental health conditions, including MDD. Psychiatrists or residents were invited to participate based on a pre-selected criterion of treating adults for any mental health conditions at their institution. The study was mainly disseminated to psychiatrists and residents by email, but other recruitment methods at the primary hospital involved disseminating study information in a weekly newsletter and at staff meetings.

### Measures

#### Introductory questions

Participants reported on their clinical experience by answering four questions (provided in Appendix 1). They indicated their job title (clinic or department head, psychiatrist, resident), the number of years they have been practicing psychiatry, and for which mental health conditions they typically saw patients. To gauge their specific expertise in treating MDD or related co-morbidities, participants also indicated how many patients with MDD or Generalised Anxiety Disorder they saw on a weekly basis.

#### Support tool ratings

Participants rated each CST on various attributes, using a five-point Likert-type scale, where higher ratings indicated more favourable perceptions. Participants rated the CST summary’s accuracy (based on information from the full clinical note), how useful it was, how confident they would feel using it in their clinical practice, and how much important information from the full clinical note it captured. For the CST recommendation, they rated how much they agreed with the recommendation and how confident they were that it was the right decision for the patient.

#### AI familiarity

Participants indicated how familiar they were with methods in AI or machine learning on a five-point scale (with higher responses indicating more familiarity).

### Experimental procedures

**Fig. 1:**
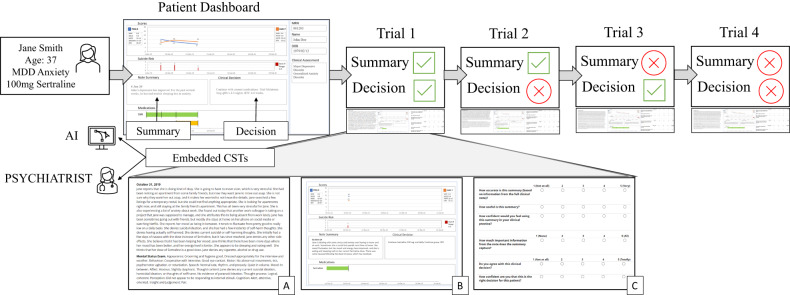
Experimental design. Psychiatrists were introduced to a hypothetical 'patient' and instructed on how to interpret information from two CSTs embedded in a patient dashboard (a note summary and clinical decision). Psychiatrists were randomised to believe the source of CSTs was either AI or a psychiatrist. All participants completed four trials in which they read a full clinical note from the patient’s “visit” (**A**), reviewed the dashboard containing CSTs related to that visit (**B**) and rated the CSTs on various attributes (**C**). Across trials, CST information differed in quality, e.g., in Trial 2, the summary was correct (contained relevant information from the full note) and the decision was incorrect (inconsistent with clinical guidelines).

The experiment was approved by our institution’s Research Ethics Board (CAMH-REB 032-2021). Psychiatrists were informed that the study 'evaluated tools being developed to facilitate the review of patient information and support clinical decision-making' and all participants provided their informed consent to complete the experiment, which was administered entirely online. Participants confirmed their eligibility by providing their institutional email address and indicating that they primarily treated adults. Next, they completed the introductory questions and started the experimental procedures (depicted in Fig. 1).

First, participants were introduced to a hypothetical patient, Jane Smith, who suffered from moderate MDD and Social Anxiety Disorder. They were informed that they would read full clinical notes describing four of Jane’s outpatient visits and review CSTs corresponding to each visit. Participants were provided instructions on how to interpret the information provided by CSTs, which were embedded within a dashboard (depicted in Appendix 2). This dashboard contained a graphical representation of Jane’s self-reported MDD and anxiety severity, suicide risk, and medication use over the four visits, as well as a summary of the clinical note and a treatment recommendation at each visit (i.e. the CSTs). Clinical notes and CSTs for each visit were shown to all participants in the same order.

Once they started the experimental procedures, participants were allocated with simple randomisation into one of two conditions (i.e. the between-subjects factor). Although information (i.e. clinical notes, dashboard and CSTs) was identical in both conditions, one group was informed that CST information was generated with AI whereas the other group was informed it was written by Jane’s treating psychiatrist. Allocation was carried out automatically, ensuring blinding during data collection. The CSTs differed in quality across the four visits (i.e. the within-subjects factor). On two visits, the information and recommendations provided by the CST was correct, in that CST summaries contained details most relevant for informing Jane’s care, and CST recommendations were consistent with clinical guidelines. On two other visits, the CST summaries contained irrelevant details from the full clinical note and CST recommendations were inconsistent with guidelines. On the first visit, both CSTs were correct. The summary contained relevant information about psychosocial stressors and medication-related improvement in symptoms, and accordingly, the recommendation was that Jane continues her current medication. On the second visit, the summary was correct, but the recommendation was incorrect; despite continued improvement related to Jane’s medication, the CST recommended a decrease in its dose. On the third visit, the recommendation was correct (i.e. prescription of a medication to address side effects), but the summary was incorrect, omitting information about these side effects and significant psychosocial stressors. Finally, on the fourth visit, both the summary and recommendation were incorrect (see Fig. 1). After completing the experiment, participants rated their familiarity with AI.

### Statistical analyses

#### Primary analyses

We generated summary and recommendation ratings for each participant on each trial by calculating means across attributes for each CST. We ran two mixed-effect models examining the impact of information source (AI or psychiatrist), information quality (correct or incorrect), and their interaction on mean ratings for each CST, specifying participants as random effects to account for repeated measures across trials. We examined the estimated marginal means, and if there was evidence of an interaction, we compared means between conditions stratified by information quality. We aimed to collect four measures from at least 70 participants to attain 90% power in detecting medium effect sizes for CST information source [12].

#### Impact of clinical expertise

Clinical expertise was based on three questions (job title, years spent practicing psychiatry and the number of patients seen per week; see Appendix 1). Based on an exploration of these responses (detailed in Appendix 3), we focused on four categories of years spent practicing psychiatry (see Table 1). We examined whether this clinical expertise interacted with information quality to impact the ratings of each CST.

**Table 1:** Descriptive information for all study participants (*n* = 83) and stratified by condition.

Variable	Full	AI	Psychiatrist	Statistical test
Sample size (*N*)	83	42	41	
Job title (*N*, %)				
Clinic/Dept Head	2 (2)	1 (2)	1 (2)	*X*^*2*^ (2) = 0.59, *p* = 0.74
Physician/Psychiatrist	33 (40)	15 (36)	18 (44)	
Resident	48 (58)	26 (62)	22 (54)	
Years practicing psychiatry (*N*, %)				
0–5	49 (59)	29 (69)	20 (49)	*X*^*2*^(3) = 4.16, *p* = 0.25
6–10	11 (13)	5 (12)	6 (15)	
11–20	8 (10)	3 (7)	5 (12)	
>20	13 (16)	4 (10)	9 (22)	
No response	2 (2)	1 (2)	1 (2)	
Regularly treated conditions (*N*, % yes)				
Depression and anxiety	74 (89)	39 (93)	35 (85)	*X*^*2*^ (1) = 0.55, *p* = 0.46
Addictions and substance use	61 (73)	32 (76)	29 (71)	*X*^*2*^ (1) = 0.10, *p* = 0.75
Mood and personality	78 (94)	39 (93)	39 (95)	*X*^*2*^ (1) = 0, *p* = 1
Schizophrenia and psychosis	70 (84)	37 (88)	33 (80)	*X*^*2*^ (1) = 0.42, *p* = 0.51
Aggression and behavioural	33 (40)	17 (40)	16 (39)	*X*^*2*^ (1) = 0, *p* = 1
Concurrent disorders	39 (47)	13 (31)	15 (37)	*X*^*2*^ (1) = 0, *p* = 0.95
Trauma and stress	58 (70)	28 (67)	30 (73)	*X*^*2*^ (1) = 0.17, *p* = 0.68
Other	4 (5)	2 (5)	2 (5)	*X*^*2*^ (1) = 0, *p* = 1
Patients with depression per week				
M (SD)	11.12 (10.69)	11.64 (13.06)	10.57 (7.57)	*W* = 785, *p* = 0.68
Median (Range)	10 (0–75)	9 (0–75)	10 (2–40)	
Familiarity with AI (*N*, %)*				
1 (Not at all)	28 (38)	15 (36)	13 (32)	*X*^*2*^(4) = 3.01, *p* = 0.56
2	24 (32)	10 (24)	14 (34)	
3	16 (22)	7 (17)	9 (22)	
4	4 (5)	2 (5)	2 (5)	
5 (Extremely)	2 (3)	2 (5)	0 (0)	
M (SD)	2.03 (1.03)	2.06 (1.17)	2 (0.90)	*W* = 668, *p* = 0.86
Median (Range)	2 (1–5)	2 (1–5)	2 (1–4)	

*Only participants who completed all four trials (*n* = 74) rated their familiarity with AI. Non-parametric hypothesis tests were used for continuous variables that were not normally distributed.

#### Impact of familiarity with AI

We ran two mixed-effect models to determine whether familiarity with AI or machine learning, and its interaction with information quality, impacted the ratings of the CSTs. In these models, AI familiarity was represented as a continuous variable, and we restricted this analysis to participants randomised to believe the source of CSTs was AI.

#### Exploratory analyses

To explore how CST type (i.e. summary or recommendation) interacted with information quality to impact ratings, we ran a model including tool type as a predictor of ratings. We also examined whether information sources interacted with resident status to impact ratings (comparing residents to psychiatrists and clinic heads). Finally, we examined the impact of information quality, source, and their interaction on responses to ratings of individual attributes (described in Support tool ratings). Due to the exploratory nature of these analyses, we did not adjust for multiple comparisons.

All models were fitted with maximum likelihood. We visually inspected histograms of residuals to ensure normality assumptions were not strongly violated and plotted model residuals against fitted values to evaluate assumptions of homogenous variance. Analyses were completed in R, Version 4.1.1. Data and code are openly available online [19].

## Results

A sample of 123 individuals read the consent form. Four individuals were not eligible (i.e. they primarily treated youth), and ten individuals did not proceed with the introductory questions. Of 109 participants who completed these questions, 16 started the study more than once. For these duplicate records, we used the first set of responses from nine individuals who completed the experiment two or more times. The remaining seven individuals started reviewing instructions on how to interpret the dashboard the first time they started the study, but they did not proceed with the experimental procedures depicted in Fig. 1. They completed the experiment on their second try, so these responses were analyzed. Within this subset, we found that two individuals were exposed to instructions for both conditions (i.e. the source of sample CSTs differed between the two instructions they reviewed; see Appendix 4). Although these participants would not have seen that CST information was the same across conditions, we completed a sensitivity analysis excluding their data to ensure their ratings were not driving our main findings (reported in Appendix 4).

Our full sample consisted of 83 participants who completed the introductory questions (69 were recruited from the primary hospital). Their descriptive information is provided in Table 1. Most participants were residents, with less than 6 years of clinical experience. Most treated patients with MDD or anxiety, but patients with other conditions were also commonly seen (e.g. schizophrenia, substance use disorders and trauma-related disorders). Most participants had little or no familiarity with methods in AI or machine learning. From the full sample, 74 participants completed the entire experiment, and descriptive characteristics between these participants and the full sample were qualitatively similar (Appendix 5). Table 1 shows that descriptive characteristics did not differ between conditions (i.e. CST information source), suggesting that randomisation was successful.

### Primary analyses

In models examining how information source, quality, and their interaction impacted ratings, the main effects of information quality emerged for both CSTs. Correct summaries were given higher ratings (*M* = 3.60, 95% CI = 3.45, 3.75) than incorrect summaries (*M* = 2.95, 95% CI = 2.80, 3.09; *r* = -0.646, SE = 0.107, *p* < 0.001). Similarly, correct recommendations were given higher ratings (*M* = 3.37, 95% CI = 3.21, 3.52) than incorrect recommendations (*M* = 1.80, 95% CI = 1.64, 1.96; *r* = −1.380, SE = 0.149, *p* < 0.001). The effect of information source was also statistically significant. CST summary ratings were higher when the perceived source was another psychiatrist (*M* = 3.48, 95% CI = 3.30, 3.66), as compared to AI (*M* = 3.07, 95% CI = 2.88, 3.25; *r* = 0.416, SE = 0.147, *p* = 0.005). This effect was also observed for CST recommendations; ratings were higher when their perceived source was another psychiatrist (*M* = 2.73, 95% CI = 2.56, 2.90), as compared to an AI (*M* = 2.44, 95% CI = 2.26, 2.61; *r* = 0.481, SE = 0.158, *p* = 0.003).

**Fig. 2:**
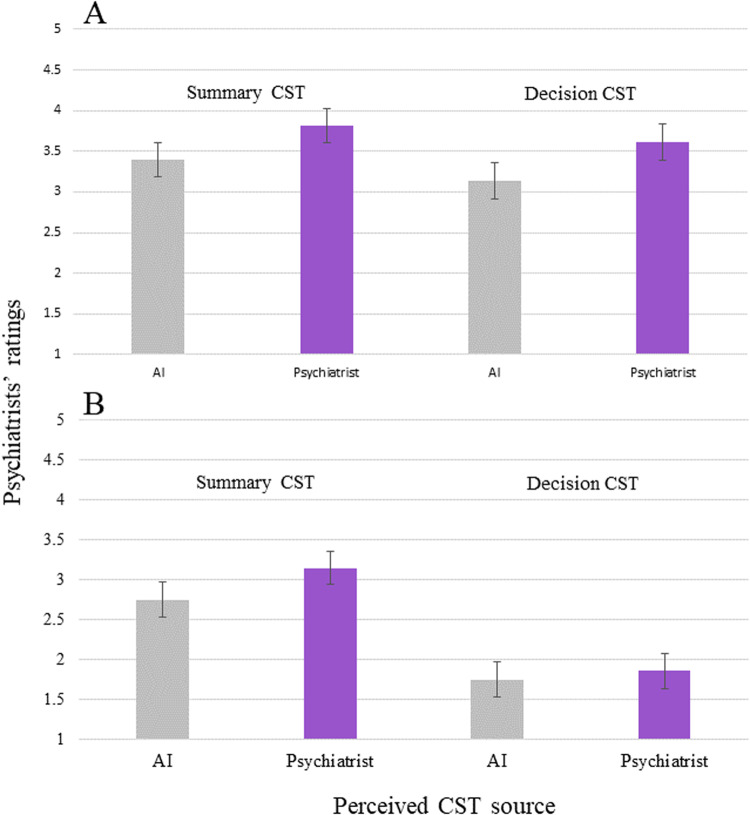
CST ratings stratified by perceived source. Ratings of each CST stratified by a perceived source on correct trials (**A**) and on incorrect trials (**B**). Note. Error bars are confidence intervals. Ratings are averaged over attributes for each CST type (i.e. four attributes for the summary and two attributes for the decision; see Table 1). All attributes were rated on a scale from 1–5, with higher scores indicating more favourable ratings.

For CST summary ratings, there was no interaction of information source and quality (*r* = −0.016, SE = 0.150, *p* = 0.914), suggesting that differences between conditions were not based on summary quality (Fig. 2A, B). There was a trend of this interaction for CST recommendation ratings, but the effect was not statistically significant (*r* = −0.369, SE = 0.209, *p* = .079). Examining the estimated mean recommendation ratings for each condition by quality, ratings were higher when the perceived source of information was another psychiatrist (*M* = 3.61, 95% CI = 3.39, 3.83), as compared to AI, for correct recommendations (*M* = 3.13, 95% CI = 2.90, 3.35; difference = −0.481, SE = 0.159, *p* = 0.003) (Fig. 2a). For incorrect recommendations, the difference between the two conditions was not statistically significant (psychiatrist *M* = 1.86, 95% CI = 1.64, 2.08; AI *M* = 1.75, 95% CI = 1.52, 1.97; difference = −0.113, SE = 0.161, *p* = 0.486) (see Fig. 2b).

Findings were not qualitatively different excluding the two participants who reviewed instructions for both conditions (reported in Appendix 4).

### Impact of clinical expertise

We examined whether years spent practicing psychiatry interacted with information quality to impact ratings for each CST (see Table 2 for results). CST ratings were higher on correct trials, and there was no impact of expertise on CST ratings. Expertise did not interact with information quality to predict CST recommendation ratings. For CST summary ratings, however, there was evidence of their interaction, for comparisons of participants practicing psychiatry for 0–5 years with those practicing for 11–20 years. For this latter group, there was no difference between summary ratings on correct and incorrect trials (0.196, SE = 0.245, *p* = 0.424), whereas this difference emerged for participants practicing psychiatry for 0–5 years (0.810, SE = 0.095, *p* < 0.001), as well as the other expertise categories (6–10 years: 0.531, SE = 0.218, *p* = 0.016; >20 years: 0.515, SE = 0.196, *p* = 0.009). However, sample sizes in individual groups were small, which could explain why summary ratings were not statistically significantly different between correct and incorrect trials in the smallest group of participants practicing psychiatry for 11–20 years (*n* = 8). In summary, there is little evidence that clinical expertise impacts CST ratings (and this is also true when expertise is represented as the number of patients with MDD or anxiety seen per week, as reported in Appendix 6).

**Table 2:** Results of examining the impact of clinical expertise and AI familiarity on CST ratings.

	CST summary	CST recommendation
	*r* (SE), *p*	*r* (SE), *p*
**Clinical expertise**		
Information quality	−0.810 (0.094), <0.001	−1.515 (0.134), <0.001
Expertise*		
6–10 years	−0.137 (0.240), 0.569	0.032 (0.250), 0.897
11–20 years	−0.112 (0.276), 0.684	0.339 (0.285), 0.235
>20 years	−0.185 (0.228), 0.419	0.174 (0.235), 0.462
Expertise × quality		
6–10 years	0.279 (0.236), 0.239	−0.011 (0.332), 0.973
11–20 years	0.613 (0.261), 0.020	−0.592 (0.369), 0.110
>20 years	0.295 (0.216), 0.173	−0.122 (0.305), 0.691
AI Familiarity**		
Information quality	–0.898 (0.223), <0.001	–1.749 (0.303), <0.001
Familiarity	–0.209 (0.097), 0.036	−0.193 (0.110), 0.083
Familiarity x Quality	0.120 (0.095), 0.207	0.192 (0.129), 0.138

Clinical expertise is represented as years practicing psychiatry.

*The reference group is 0–5 years; **Analyses restricted to participants randomised to believe the source of CSTs was AI.

### Impact of familiarity with AI

We examined whether familiarity with AI or machine learning interacted with information quality to impact CST ratings for participants randomised to believe their source was AI (results are provided in Table 2). For both CSTs, ratings were higher on correct trials than on incorrect trials. Participants who were more familiar with AI tended to rate CST summaries less favourably, but this association was not statistically significant for CST recommendation ratings. However, few participants reported being familiar with these methods, with only four psychiatrists providing ratings over 3 on a 5-point scale. Familiarity also did not interact with information quality to impact ratings of either CST.

### Exploratory analyses

#### CST type

In a model examining the impact of information quality, CST type, and their interaction on ratings, main effects emerged for information quality (*r* = −1.567, SE = 0.090, *p* < 0.001) and CST type (*r* = 0.231, SE = 0.089, *p* = 0.010). Summary ratings were higher (*M* = 3.27, 95% CI: 3.14, 3.41) than recommendation ratings (*M* = 2.59, 95% CI: 2.46, 2.71), but this effect also interacted with information quality (*r* = 0.910, SE = 0.127, *p* < 0.001). Ratings for both CSTs were higher when they provided correct information (summary, *M* = 3.60, 95% CI: 3.44, 3.76; recommendation, *M* = 3.37, 95% CI: 3.21, 3.53) as compared to an incorrect information (summary, *M* = 2.95, 95% CI: 2.79, 3.11; recommendation, *M* = 1.80, 95% CI: 1.64, 1.96). However, differences based on quality between the two CST types were larger on incorrect trials (difference = −1.141, SE = 0.091, *p* < 0.001), as compared to correct trials (difference = −0.231, SE = 0.090, *p* = 0.010). This interaction (depicted in Appendix 7) suggests that less favourable perceptions of incorrect trials are more pronounced for CST recommendations than summaries.

#### Resident status

According to our primary results, participants rated CSTs more favourably if their perceived source was another psychiatrist as compared to AI. This effect may reflect that psychiatrists are hesitant or unwilling to criticise information generated by a potential colleague (i.e. another psychiatrist). If this is true, this hesitation may be more pronounced in residents than in established clinicians (i.e. psychiatrists, physicians or clinic heads). To evaluate this possibility, we examined whether job title interacted with information source to impact CST ratings. In this model, there was an effect of information source (*r* = 0.384, SE = 0.177, *p* = 0.033), and ratings did not differ between residents and non-residents (*r* = 0.053, SE = 0.166, *p* = 0.750). Resident status also did not interact with information source (*r* = −0.076, SE = 0.227, *p* = 0.869), suggesting that residents did not rate the CSTs differently based on whether they believed their source was AI or another psychiatrist.

#### Ratings of individual attributes

**Fig. 3:**
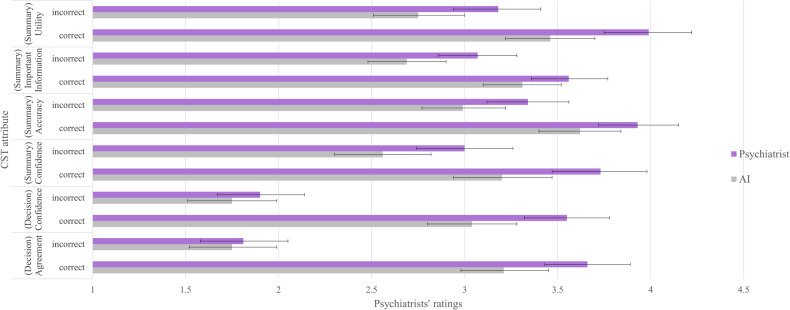
Psychiatrists’ ratings of individual CST attributes, stratified by information quality (correct and incorrect) and source (AI and Psychiatrist). Note. Error bars are confidence intervals. All attributes were rated on a scale from 1–5, with higher scores indicating more favourable ratings.

Results for the impact of information quality, source, and their interaction on ratings for each CST attribute are provided in Appendix 8. Mean ratings, stratified by condition, are depicted in Fig. 3. Not surprisingly, attribute ratings were higher for both CSTs on correct trials, but the effect of information source varied between attributes. This effect was largest for the rated utility of the summary, agreement with the recommendation, and confidence in using the two CSTs. The effect was smaller for ratings reflecting the summary’s accuracy and not statistically significant for ratings reflecting the inclusion of important information from the full clinical note (Appendix 8). Consistent with our primary findings, the effect of information source was statistically significant on correct trials for ratings of both recommendation attributes (i.e. agreement and confidence), but it was not statistically significant on incorrect trials.

## Discussion

Our experiment examined how psychiatrists respond to information provided by CSTs, focusing on their quality (as providing correct or incorrect information) and perceived source (AI or a psychiatrist). Based on promising avenues for developing CSTs for treating MDD [1, 9], we examined perceptions of clinical note summaries and treatment recommendations. Not surprisingly, ratings for both CSTs were higher when they provided correct information. Interestingly, psychiatrists rated CSTs less favourably when they believed their source was AI, as compared to another psychiatrist. Because CST information was identical in both conditions, this finding suggests that psychiatrists in our study prefer information generated by human experts (i.e. other psychiatrists) over AI systems. For CST recommendation ratings, however, this preference did not emerge when recommendations were incorrect. In contrast with prior studies [17, 18], we found limited evidence that clinical expertise or familiarity with AI moderated the impact of information quality on ratings; differences in ratings on correct and incorrect trials did not depend on how many years participants practiced psychiatry or how familiar they were with AI. However, these conclusions are limited by the small sample sizes for clinical expertise groups and little variability in AI familiarity.

Our primary finding that psychiatrists may be biased against information derived from AI is surprising, given positive perceptions of AI-based CSTs for treating MDD and other health conditions noted in prior work [14, 15]. Our study addresses a limitation of this research by contrasting perceptions of AI and human-derived information. Although psychiatrists may have a general openness toward integrating CSTs into clinical practice, they may be sceptical or critical of this information when it is AI-based. This finding is also consistent with research into patients’ perceptions of CSTs, which finds that patients are less confident in AI-assisted interpretations of injuries from radiographs than in interpretations of clinicians [20]. A preference for human-based information has emerged in studies of general samples as well; participants deciding how to allocate hospital resources or humanitarian aid rated advice provided by humans as being more expert and useful than advice provided by AI [21]. However, perceptions of AI change over time [22]. Our findings may reflect current concerns about the performance of AI-based CSTs in real-world settings [4] or their limited applicability in psychiatry due to the interpersonal nature of mental health care [13]. Perceptions may change as AI applications in healthcare improve and with corresponding changes in public perception.

For CST recommendation ratings, the source of information interacted with its quality (i.e. there was no preference for psychiatrist-derived recommendations on incorrect trials), whereas ratings were higher for psychiatrist-generated summaries, regardless of their quality. This discrepancy in findings between CST types may be related to differences in the potential negative impacts of consulting incorrect clinical note summaries and recommendations. An irrelevant summary may not be too detrimental, especially if other information is available, such as the patient’s symptom severity or prescribed medications depicted in the dashboard psychiatrists reviewed in our experiment (Fig. 1). Consulting a treatment recommendation that is inconsistent with clinical guidelines, however, could sway psychiatrists toward providing improper care, which would negatively impact a patient’s health and wellbeing. This explanation is consistent with findings from our exploratory analysis, which suggested that incorrect treatment recommendations were rated less favourably than incorrect summaries. Alternatively, findings could relate to difficulties evaluating the quality of information between the two CST types. This evaluation may be more challenging for a clinical note summary, given that psychiatrists may not always agree on which information about patients is most relevant for informing care. In contrast, there may be a clearer distinction between correct and incorrect treatment recommendations, given that psychiatrists are well-versed in standardised guidelines for treating MDD.

Our experiment provided little evidence that clinical expertise interacted with information quality to impact ratings, which does not support findings that inexperienced physicians are more likely to rely on incorrect diagnostic advice, as compared to experts [17]. The psychiatrists in our study provided less favourable ratings of incorrect CST information, regardless of how many years they practiced psychiatry. The difference between summary ratings on correct and incorrect trials was not statistically significant for one expertise group (practicing psychiatry for 11–20 years), differing from the group with the largest difference (practicing for 0–5 years). However, this interaction likely emerged due to the former group being under-powered to detect a difference. We focused on interactions of clinical expertise with information quality, due to concerns that less experienced clinicians might be more susceptible to incorrect information, but we also considered interactions with information source. Specifically, younger psychiatrists might be more ‘technologically-minded’ and perceive AI-based information more favourably than older psychiatrists, which was observed in a study of patients [20]. When using job titles as a proxy for age, there was no evidence that residents (which tend to be younger) rated AI-based information more favourably than other clinicians. Overall, findings from our experiment suggest that less experienced or younger psychiatrists are not any more susceptible to relying on incorrect or AI-based information.

In our analysis examining the impact of familiarity with AI on CST ratings, psychiatrists who reported being more familiar with such methods rated AI-based summaries less favourably. This association is consistent with prior research, since psychiatrists more familiar with AI were less likely to follow AI-based treatment recommendations for MDD in another experiment [18]. Having some exposure to or understanding of methods in AI and machine learning may contribute to an awareness of their limitations, which could have influenced psychiatrists’ ratings.

Although we did not design our experiment to examine why ratings of CSTs differ based on their perceived source, findings from our exploratory analyses provide some insight. We examined whether a preference for psychiatrist-generated information emerged due to a reluctance to criticise the opinions of a colleague, by examining whether this preference was more pronounced in residents, who may be more susceptible to such social pressures Although this was not found, the analysis does not rule out the possibility that social pressures influenced ratings, rather than unfavourable perceptions about AI. Furthermore, the human expert comparison in our experiment was described as the patient’s treating psychiatrist, which suggests a degree of expertise related to familiarity with the patient. This expertise would not be accessible to AI, but it may be particularly important given the interpersonal nature of MDD assessment and care [13].

Our exploratory analysis of individual CST attributes nevertheless suggests that a preference for psychiatrist-generated CST information may be related to the use of heuristics. This preference was less pronounced for ratings of the summary’s accuracy and its inclusion of important information. Ratings for these attributes may have required a closer review of the summary’s information and a comparison with the full clinical note. This deeper review or reflection may have also been required for evaluating incorrect clinical recommendations, for which information source did not impact ratings. In contrast, psychiatrists may have relied on heuristic or intuitive thinking when rating their agreement with correct treatment recommendations, the utility of summaries, and their confidence in using both CSTs (Figs. 2a, 3). According to a dual-process theory of cognition, limited cognitive resources prevent individuals from engaging in deep or analytical thinking unless necessary, which may contribute to the development of general heuristics about AI performance [23]. Although the use of heuristics has been proposed to contribute to an over-reliance on AI systems [23], we observed the opposite effect, where heuristics may have prompted a bias against AI-based information.

### Limitations

Our ability to address our secondary objective was limited by little variability in clinical expertise and familiarity with AI or machine learning, and any conclusions related to these factors should be interpreted with caution. Additionally, visits were presented to psychiatrists in the same order, to preserve the temporal progression of the patient’s care. However, this introduces the possibility of order effects, since the quality of information was manipulated in the same way across visits for all participants. We found that psychiatrists rated incorrect information provided by the CSTs less favourably than correct information, which is consistent with prior findings that psychiatrists find incorrect antidepressant recommendations less useful than correct ones. Despite this perception, psychiatrists in that study made less accurate treatment decisions when presented with incorrect recommendations [18]. Because we did not examine psychiatrists’ behaviours, it is unclear whether exposure to incorrect CST information would have impacted treatment decisions or clinical care. This point underscores another limitation of our experiment; psychiatrists provided ratings in the context of artificial and controlled scenarios, which may not generalise to their perceptions in real-world clinical settings.

### Future directions

Future research should measure the impact of CSTs on perceptions and behaviours in more ecologically valid contexts. To bypass ethical concerns related to measuring their impact on patients, simulated settings can be used, involving actors or virtual reality to recreate clinical encounters [15]. This research will be necessary to investigate the downstream implications of integrating AI into psychiatric care. Additionally, the use of heuristic thinking may have contributed to the effect of information source in our experiment, but this explanation is based on exploratory analyses of individual attributes and should be explored further (e.g. in qualitative interviews with psychiatrists). If this explanation is supported, future research might focus on implementing cognitive strategies to promote analytical or critical thinking when engaging with AI-based CSTs. A variety of interventions have been developed to promote this thinking style among physicians, with some evidence favouring the use of cognitive forcing strategies [24]. Such strategies involve delaying the presentation of CST information or making initial decisions without CST assistance, and they can reduce an over-reliance on incorrect AI-based recommendations [23]. However, how such strategies might impact decision-making for psychiatric care has not been explored. Furthermore, many factors can impact analytical thinking in clinical settings. The capacity to critically engage with AI-based information may be impacted by competing attentional demands in a busy environment or trait differences in a need for cognition [23]. Even a psychiatrist’s emotional state may be influential [25], given associations between heuristic thinking and anger or irritability [26]. Further research should explore the various factors impacting psychiatrists’ perceptions and behaviours related to AI-based CSTs, as well as downstream impacts on patient care. This research will be critical to support the future integration of AI-based information into clinical decision support in psychiatry.

## Supplementary information

Supplemental Appendices
