# Supplementary material for: Out with AI, in with the psychiatrist: a preference for human-derived clinical decision support in depression care
Source: Transl Psychiatry. 2023 Jun 16;13:210. doi: 10.1038/s41398-023-02509-z (PMC10275935; doi:10.1038/s41398-023-02509-z)
Supplement: Supplementary file 1 — Supplemental Appendices [file 41398_2023_2509_MOESM1_ESM.docx]

**Out with AI, in with the psychiatrist:
A preference for human-derived clinical decision support in depression care**

**Supplemental Appendices**

**Appendix 1**

***Introductory questions***

*For CAMH psychiatrists:* What is your job title at CAMH?
*For psychiatrists at other institutions:* Which of the following best describes your job title?

o Clinic/Department Head
o Physician
o Psychiatrist
o Resident

How many years have you been practicing psychiatry, including residency? (Please select one option)

o 0-5 years
o 6-10 years
o 11-20 years
o over 20 years

Which mental health condition(s) do you typically see patients with?

o Addictions/Substance Use
o Anxiety & Depression
o Mood & Personality Disorders
o Schizophrenia & Psychosis
o Aggression & Behavioural Issues
o Concurrent Disorders
o Trauma & Stress Disorders
o Other
If other, please specify: ________________________________________

How many patients with Major Depressive Disorder (MDD) or Generalized Anxiety Disorder (GAD) do you see in an average week?_________
*(response range set to 0-100)*

**Appendix 2**
 ***Study Instructions***
After completing the introductory questions (provided in Appendix 1), participants were provided with the instructions to complete the experimental trials (provided below).

We are currently evaluating some clinical summarization and decision-making tools, and we would like your help!

You will be asked to read 4 clinical notes describing Jane Smith, a 37-year-old female patient. Jane has been diagnosed with moderate, single episode Major Depressive Disorder and Social Anxiety Disorder (social phobia). She is currently taking Sertraline (100mg daily). For each note you read, you will be presented with a dashboard depicting information about Jane.

The dashboard will include scores from the PHQ-9 (depression) measure and the GAD-7 (anxiety) measure completed by the patient to-date, as well as their severity ranges.

The dashboard will also include suicide risk scores, which refer to the 9^th^ (suicidal ideation) item on the PHQ-9. These scores range from 0-3.

The dashboard will also show the medications prescribed to the patient to-date.


*For participants assigned to the AI condition:*
Importantly, the dashboard will contain a summary of the most recent clinical note generated with artificial intelligence (AI), as well as the AI’s clinical decision.

*For participants assigned to the psychiatrist condition:*
Importantly, the dashboard will contain a summary of the most recent clinical note written by the treating psychiatrist, as well as the psychiatrist’s clinical decision.

You will be asked 4 questions about this summary and 2 questions about this clinical decision. Your ability to judge this case may be limited, but please do your best to answer the questions using the information provided.

***Experimental trials***
On four trials, participants read a full clinical note and they reviewed a patient-level dashboard with two embedded CSTs for each of Jane’s visits. The full notes and dashboards (with embedded summaries and clinical decisions) for each trial are provided below.

*Trial 1*

Read the first note below. **October 31, 2019**

Jane reports that she is doing kind of okay. She is going to have to move soon, which is very stressful. She had been renting an apartment from some family friends, but now they want Jane to move out asap. She is not sure why they want her out asap, and it makes her worried to not know the details. Jane searched a few listings for a temporary rental, but she could not find anything appropriate. She is looking for apartments right now, and still staying at the family friend’s apartment. This has all been very stressful for Jane. She is also experiencing a lot of anxiety about work. She found out today that another work colleague is taking on a project that Jane was supposed to manage, and she attributes this to being absent from work lately. Jane has been sometimes going out with friends, but mostly she stays at home on her phone on social media or watching Netflix. She reports her mood as being in between. It tends to fluctuate from pretty good to really low on a daily basis. She denies suicidal ideation, and she has had a few incidents of self-harm thoughts. She denies having actually self-harmed. She denies current suicidal or self-harming thoughts. She initially had a few days of nausea with the dose increase of Sertraline, but it has since resolved. Jane denies any other side effects. She believes that it has been helping her mood. Jane thinks that there have been more days where her mood has been better, and her energy level is better. She appears to be sleeping and eating well. She thinks that her dose of Sertraline is a good dose. Jane denies any cigarette, alcohol or drug use.

**Mental Status Exam.** Appearance: Grooming and hygiene good. Dressed appropriately for the interview and weather. Behaviour: Cooperative with interview. Good eye contact. Motor: No abnormal movements, tics, psychomotor agitation, or retardation. Speech: Normal rate, rhythm, and prosody. Quiet in volume. Mood: In between. Affect: Anxious. Slightly dysphoric. Thought content: Jane denies any current suicidal ideation, homicidal ideation, or thoughts of self-harm. No evidence of paranoid ideation. Thought process: Logical, coherent. Perception: Did not appear to be responding to internal stimuli. Cognition: Alert, attentive, oriented. Insight and judgement: Fair.

Next, review the dashboard on the next page and answer the questions. Feel free to return to this page to review the note again, if needed.

*Trial 2*

Read the second note below. **November 26, 2019**Jane reports that she is preparing to move in with her sister temporarily, as rent at her current apartment has become too much for her to manage. She also reports that she has been really stressed out lately because her paternal grandfather has pneumonia, and he is in the hospital with cancer. Jane is also going through a busy period at work. She is concerned that she is very behind with tasks, given she has been absent lately due to the family health difficulties. Jane denies any suicidal ideation. She has had some thoughts to self-harm by cutting about twice a week, but denies any action taken or intent to act on these thoughts. She denies any homicidal ideation. Jane continues to take Sertraline daily and denies any side effects from the medication. She feels that her eating and sleep have regulated and attributes this to the Sertraline. Jane thinks that it has improved her anxiety level “a little bit”. For instance, Jane participated in a work group project and she says that in the past, she would have asked her supervisor to work alone. Although she didn’t say or contribute much, working in her group went okay. She has been taking the Sertraline at 100 mg daily for at least 6 weeks.

**Mental Status Exam.** No significant change. Appearance: Grooming and hygiene good. Dressed appropriately for the interview and weather. Behaviour: Cooperative with interview. Good eye contact. Motor: No abnormal movements, tics, psychomotor agitation or retardation. Speech: Normal rate, rhythm, and prosody. Quiet in volume. Mood: Really stressed. Affect: Anxious and dysphoric, but less so than previously. Thought content: Jane denies any current suicidal ideation, homicidal ideation or thoughts of self-harm. No evidence of paranoid ideation. Thought process: Logical, coherent. Perception: Denied auditory or visual hallucinations. Did not appear to be responding to internal stimuli. Cognition: Alert, attentive, oriented. Insight and judgment: Fair.

Next, review the dashboard on the next page and answer the questions. Feel free to return to this page to review the note again, if needed.

*Trial 3*

Read the third note below.

**December 16, 2019**

Jane reports that she is doing okay. She has been feeling stressed out at work because her team is nearing the end of a project and there are many tasks to complete. She is finding it somewhat overwhelming because she has recently missed some days of work and has not encountered work stress for a while. Jane received good feedback from her supervisor today; he noted that her performance has improved but mentioned that she needs to focus more. Jane says work has been easier to come into because people disrupt her less at the office. Aside from work stress, Jane says that she has been feeling really emotional lately. She says that there have been arguments between her sister, mother, and grandmother. She says that there is constant conflict between family members, and it is so common, that they sometimes will all scream at each other. This has been difficult to adjust to because Jane had been so used to living on her own, and there was minimal fighting when she was not living with her sister. Jane says that she is still trying to find a place to move into, but she needs to put aside some money to do it. She feels that her anxiety level has been okay. She has not had any significant exacerbations of her anxiety or panic attacks. She denies any safety risks, including no suicidal ideation or thoughts of self-harm. She continues to take the Sertraline now at 125 mg oral daily. However, she says that for the past few days, she has been experiencing a lot of reflux and some nausea.

**Mental Status Exam Appearance:** Grooming and hygiene good. Dressed appropriately for the interview and weather. Behaviour: Cooperative with interview. Good eye contact. Motor: No abnormal movements, tics, psychomotor agitation, or retardation. Speech: Normal rate, rhythm, and prosody. Quiet in volume. Mood: Okay. Affect: Slightly dysphoric and slightly anxious. Thought content: Jane denies any current suicidal ideation, homicidal ideation or thoughts of self-harm. No evidence of paranoid ideation. Thought process: Logical, coherent. Perception: Did not appear to be responding to internal stimuli. Cognition: Alert, attentive, oriented. Insight and judgement: Fair.

Next, review the dashboard on the next page and answer the questions. Feel free to return to this page to review the note again, if needed.

*Trial 4*

Read the fourth note below.

**January 7, 2020**

Jane reports that she has been feeling very sad and anxious for the past few days. She has not had any panic attacks. Jane says that she has had an argument with her sister over her finances and attendance at work, resulting in current tension at home. She reported that she is also stressed about a big work deadline this week and having to complete final tasks for a project after having missed a few days of work. Jane says that she was not home much over the holidays because she was out a lot with friends and work colleagues. She believes her sister likes that Jane is getting out more. Jane was home with her sister’s family on Christmas. She says that it was weird because her grandfather would have usually been there in the past, but he had recently passed, and was not there this year. She feels that, as a result, it was a hard day. Jane is unsure how her grandmother is doing, as she hasn't really talked much to her due to some family conflict. She is unsure of her plan to move out of her sister’s home. She denies any suicidal ideation. She continues to have the usual self-harming thoughts such as to cut herself as a way to relieve stress and pain. She manages to push the self-harming thoughts aside by going out or going to sleep. She has not had any actual intent to self-harm and had not made any steps toward self-harming. She says that she is not sure if she is open to continue going to therapy. She says that she is not sure that she would actually commit to going. Jane continues to take the Sertraline 125 mg oral daily, plus Ranitidine 150 mg oral daily 1 hour prior to taking the Sertraline. She does feel that it has helped her heartburn, but there was a day or two where she did still experience heartburn. Jane continues to feel that the antidepressant has been helpful. She denies any other side effects.

**Mental Status Exam.** Appearance: Grooming and hygiene good. Dressed appropriately for the interview and weather. Behaviour: Cooperative with interview. Good eye contact. Motor: No abnormal movements, tics, psychomotor agitation, or retardation. Speech: Normal rate, rhythm, and prosody. Quiet in volume. Mood: “very sad" and anxious. Affect: Slightly dysphoric and slightly anxious. Thought content: Jane denies any current suicidal ideation, or homicidal ideation. Endorses chronic suicidal thoughts with no intent or plan to harm herself. No evidence of paranoid ideation. Thought process: Logical, coherent. Perception: Did not appear to be responding to internal stimuli. Cognition: Alert, attentive, oriented. Insight and judgement: Fair.

Next, review the dashboard on the next page and answer the questions. Feel free to return to this page to review the note again, if needed.

***CST ratings***
At each of the four trials, participants answered the same four questions about the summary and the same four questions about the decision. An example of how these questions were displayed to participants (for trial 1) are provided below.

**Appendix 3**

***Explorations of clinical expertise***

Since few clinic or department heads took part (*n*=2), we grouped them with psychiatrists to make job title a binary factor (i.e., 48 residents vs. 35 psychiatrists, which included clinic heads). Most residents (*n*=46, 96%) had 0-5 years of experience, whereas few psychiatrists were represented in this category (n=3, 9%). This is not surprising, since residency lengths in Canada for psychiatry are typically 5 years. The categories representing years of experience were more diverse for psychiatrists and clinic heads; many had >20 years of experience (*n*=13, 37%), followed by 6-10 years (*n*=10, 29%), then 11-20 years (*n*=8, 23%), and fewest had 5 or less (*n*=3, 9%). The years spent practicing psychiatry differed significantly between residents and psychiatrists, *X*^2^(3)=65.70, *p*<.001, and thus, we concluded that job title provided information on clinical expertise that was redundant with years spent practicing psychiatry, and we excluded job title from this analysis.

We did not find that psychiatrists reported seeing more patients with MDD than residents (psychiatrists: M=14.03, SD=14.62, residents: M=9.06, SD=6.03; *W*=944, *p*=.225). At the same time, the number of patients seen may not be the best indicator of expertise, since more experienced psychiatrists or clinic heads may treat fewer, more complex cases, or dedicate more time toward administration, leadership, clinical training, and/or research. Thus, we focused on years spent practicing psychiatry as our main indicator of clinical expertise (reported in the Results). However, we retained number of patients with MDD or anxiety seen weekly as an alternative indicator of clinical expertise to years spent practicing psychiatry (reported in Appendix 6).

Since most (~90%) of participants regularly treated patients with MDD or anxiety, we did not examine whether ratings differed for participants who treated patients with other mental health conditions.

**Appendix 4

*Sensitivity analysis excluding participants exposed to instructions for both conditions***

As described in the Results, 16 individuals started the study more than once. For these duplicate records, 8 individuals completed the experiment two or more times, and we only included their responses from the first time they completed the study. One individual completed three experimental trials the first time they started the study, and only the introductory questions a second time; for this participant, we included responses from their first completion as well.

All remaining seven individuals completed the introductory questions the first time they started the study, and they started reviewing the study instructions (provided in Appendix 2); however, they did not proceed to the experimental procedures depicted in Figure 1 (i.e., they were not introduced to the hypothetical patient, they did not read any of the full clinical notes, and they did not review any CST information). The second time they started the study, they completed the introductory questions, reviewed instructions, and completed the experimental procedures. For two of these participants, the source of CSTs in the instructions they reviewed the first time was the same as the second time. For the remaining five participants, it was different. However, three of these five participants did not finish reading the instructions, and they did not view the final webpage of the instructions which indicated the source of the CST. Thus, these participants would not have been exposed to instructions for both conditions. The remaining two participants viewed the final webpage, which indicated the CST source, so they knew that CSTs in the experiment had two different sources. These participants would not have known that the information from the two sources was the same (since they did not proceed with the experimental procedures the first time they started the study). Nevertheless, we repeated our primary analysis excluding their data, in case the knowledge that CSTs had different sources biased their responses at the experimental trials.

We ran the two mixed effect models from our primary analysis, examining the impact of information source (i.e., AI or psychiatrist), information quality (i.e., correct or incorrect), and their interaction on mean ratings for each CST. In this analysis, we excluded data from the two participants who were exposed to instructions for two different conditions (described above).

For CST summary ratings, there was an effect of information quality (*r*= -0.717, SE=0.110, *p*<.001), information source (*r*=0.417, SE=0.151, *p*=.007), but no evidence of their interaction (*r*=-0.001, SE=0.154, *p*=.993). For CST recommendation ratings, there was also an effect of information quality (*r*=-1.345, SE=0.154, *p*<.001), information source (*r*=0.482, SE=0.158, *p*=.003), and an interaction trend (*r*=-0.411, SE=0.220, *p*=0.056). Examining differences in CST recommendation ratings between the two conditions stratified by information quality, the difference in ratings on correct trials is statistically significant (-0.482, SE=0.159, *p*=.003), whereas this difference is not statistically significant on incorrect trials (-0.071, SE=0.166, *p*=.671). Our primary findings do not qualitatively change when data from the two participants are removed, suggesting that their exposure to instructions for both conditions were not driving our main findings.

**Appendix 5**
 ***Descriptive information for the full sample, and participants who completed all four trials***

|  | Sample | |
| --- | --- | --- |
| Variable | Full (n=83) | Completers (n=74) |
| Job title (N, %) |  |  |
| Clinic/Dept Head | 2 (2%) | 2 (3%) |
| Physician/Psychiatrist | 33 (40%) | 27 (36%) |
| Resident | 48 (58%) | 45 (61%) |
| Years practicing Psychiatry (N, %) |  |  |
| 0-5 | 49 (59%) | 46 (62%) |
| 6-10 | 11 (13%) | 8 (11%) |
| 11-20 | 8 (10%) | 7 (9%) |
| >20 | 13 (16%) | 11 (15%) |
| No response | 2 (2%) | 2 (3%) |
| Regularly treated conditions (N, % yes) |  |  |
| Depression + Anxiety | 74 (89%) | 65 (88%) |
| Addictions + Substance use | 61 (73%) | 52 (70%) |
| Mood + Personality | 78 (94%) | 70 (95%) |
| Schizophrenia + Psychosis | 70 (84%) | 63 (85%) |
| Aggression + Behavioural | 33 (40%) | 28 (38%) |
| Concurrent Disorders | 39 (47%) | 34 (46%) |
| Trauma + Stress | 58 (70%) | 50 (68%) |
| Other | 4 (5%) | 4 (5%) |
| Patients with depression per week |  |  |
| M (SD) | 11.12 (10.69) | 10.91 (10.62) |
| Median (Range) | 10 (0-75) | 8 (0-75) |
| Familiarity with ML/AI (N, % yes) |  |  |
| 1 (Not at all) | - | 28 (38%) |
| 2 |  | 24 (32%) |
| 3 |  | 16 (22%) |
| 4 |  | 4 (5%) |
| 5 (Extremely) |  | 2 (3%) |
| M (SD) | - | 2.03 (1.03) |
| Median (Range) | - | 2 (1-5) |

**Appendix 6**

***Analyses of clinical expertise represented as number of patients with MDD or anxiety seen per week***
In the model representing clinical expertise as number of patients with MDD or anxiety seen per week, CST ratings were higher on correct trials. Participants who reported seeing more patients with MDD or anxiety were less likely to provide favourable ratings of the CST summary, but this association was not statistically significant for CST recommendation ratings (see the Table below). There was also no evidence of an interaction between number of patients seen and information quality on CST ratings.

*Results examining the impact of clinical expertise on CST ratings*

|  | Summary | Recommendation |
| --- | --- | --- |
|  | *r* (SE), *p* | *r* (SE), *p* |
| Information correctness | -0.723 (0.108), <.001 | -1.579 (0.152), <.001 |
| Clinical expertise | -0.021 (0.007), .005 | -0.013 (0.008), 0.083 |
| Expertise x Correctness | 0.006 (0.007), .399 | 0.000 (0.010), .977 |

*Note.* Clinical expertise as number of patients with MDD or anxiety seen per week.

Plotting the number of patients seen against summary and decision ratings to examine the linearity of associations (see plots below), the inverse associations depend on ratings across trials from one participant seeing more than 60 patients per week. With data from this participant removed, the impact of clinical expertise is no longer statistically significant for CST summary ratings (*r*=-0.01, SE=0.010, *p*=.164), and it is less pronounced for CST recommendation ratings (*r*=-0.013, SE=0.011, *p*=.239). In summary, results from these additional analyses provide limited evidence that clinical expertise, when represented as number of patients with MDD or anxiety seen per week, impacted CST ratings.

*CST Summary Ratings*


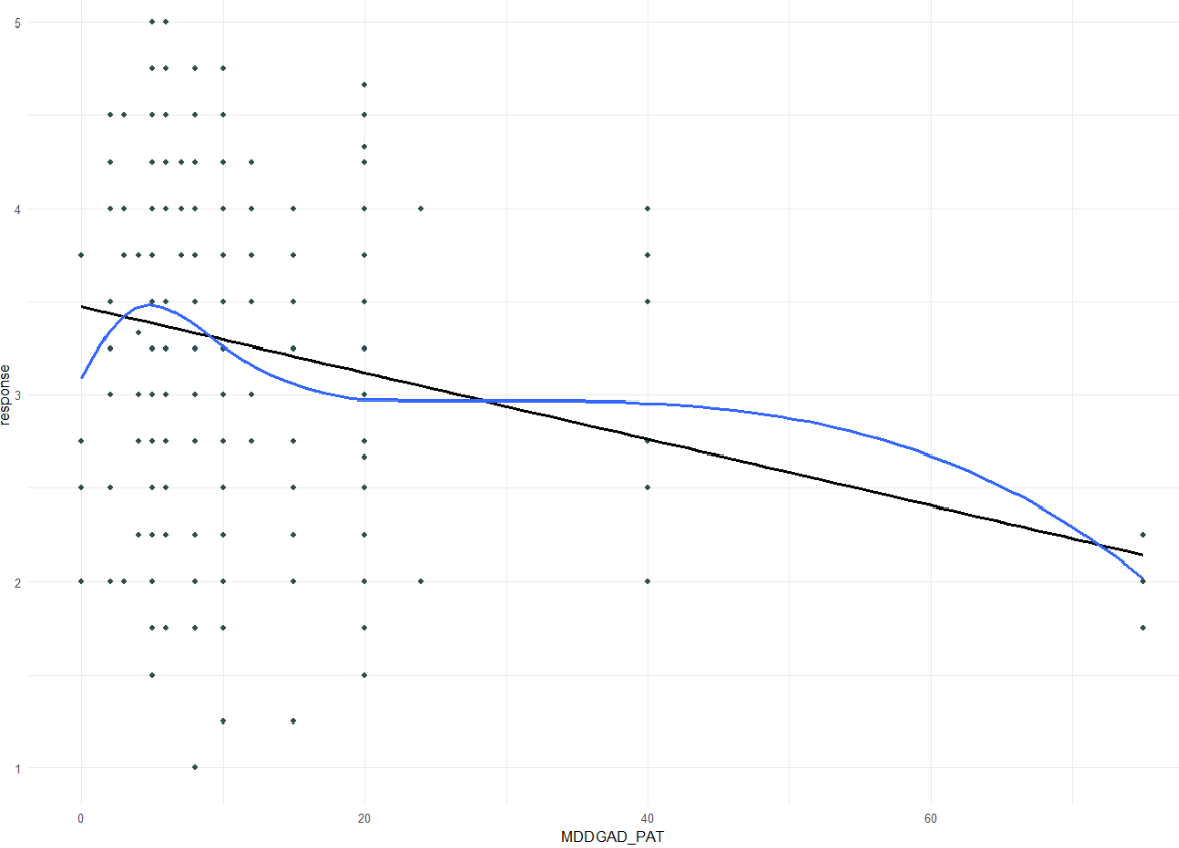


*CST Recommendation Ratings*


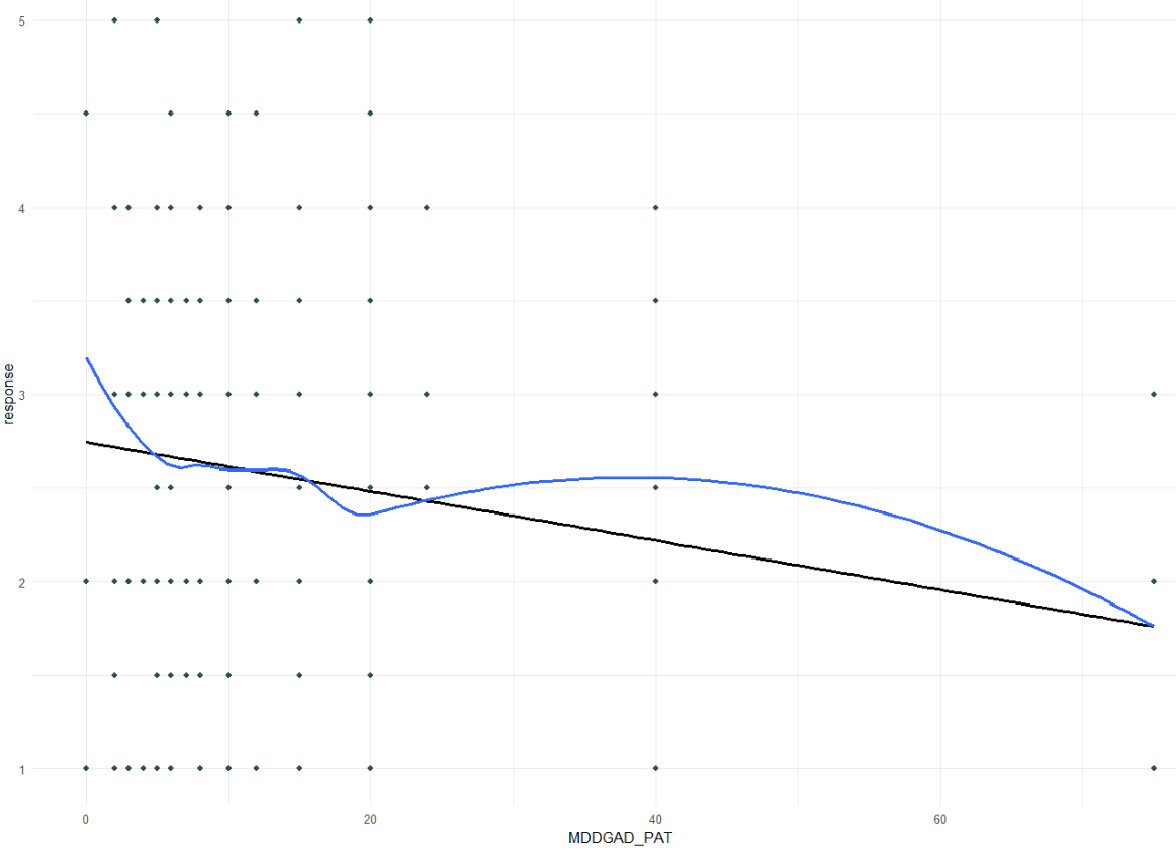


**Appendix 7** *CST ratings, stratified by CST type and information quality*


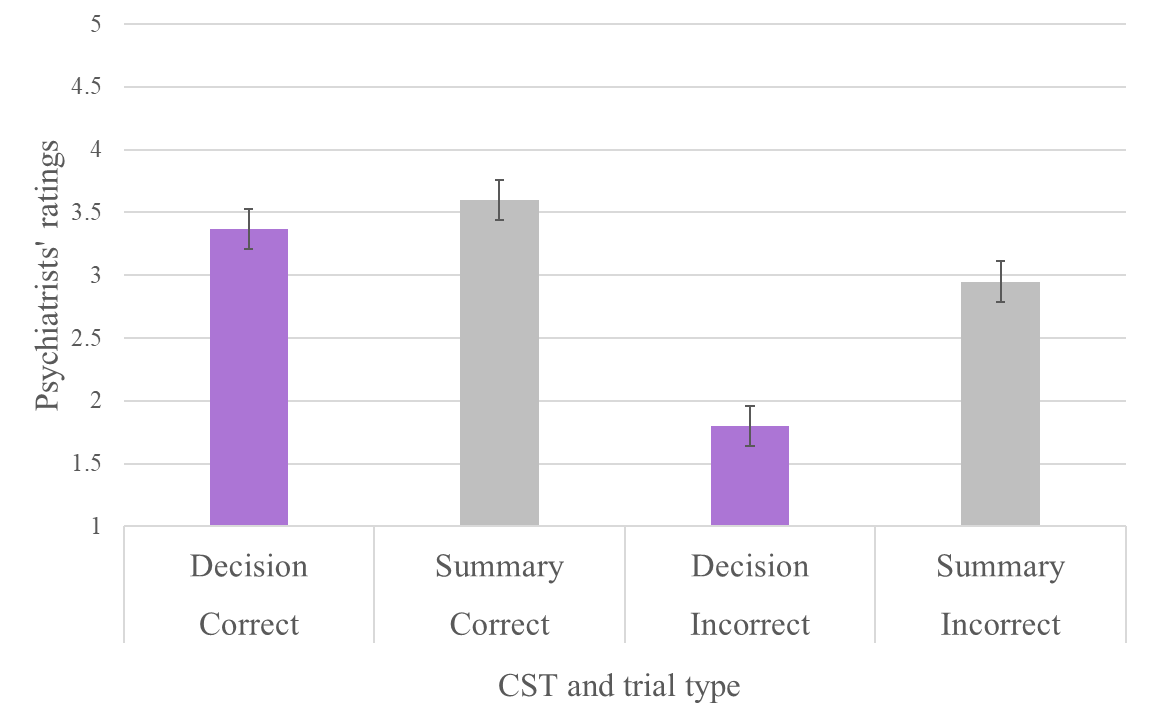


*Note.* Error bars are confidence intervals. Ratings are averaged over attributes for each CST type (i.e., 4 attributes for the summary, 2 attributes for the decision; see Table 1). All attributes were rated on a scale from 1-5, with higher scores indicating more favourable ratings.

**Appendix 8**

***Impact of information quality, source, and their interaction, on individual attribute ratings***

| *Rating* | *Source* | *Quality* | *Interaction* |
| --- | --- | --- | --- |
| How accurate is this summary (based on information from the full clinical note)? | *r*=0.314, SE=0.155, *p*=.045 | *r*=-0.627, SE=0.127, *p*<.001 | *r*=0.03, SE=0.178, *p*=.862 |
| How useful is this summary? | *r*=0.531, SE=0.168, *p*=.002 | *r*=-0.704, SE=0.120, *p*<.001 | *r*=-0.108, SE=0.167, *p*=.520 |
| How confident would you feel using this summary in your clinical practice? | *r*=0.524, SE=0.183, *p*=.005 | *r*=-0.645, SE=0.128, *p*<.001 | *r*=-0.085, SE=0.179, *p*=.638 |
| How much important information from this note does the summary capture? | *r*=0.254, SE=0.148, *p*=.087 | *r*=-0.617, SE=0.113, *p*<.001 | *r*=-0.119, SE=0.160, *p*=.455 |
| Do you agree with this clinical decision? | *r*=0.448, SE=0.165, *p*=.007 | *r*=-1.460, SE=0.161, *p*<.001 | *r*=-0.388, SE=0.225, *p*=.087 |
| How confident are you that this is the right decision for the patient? | *r*=0.510, SE=0.166, *p*=.002 | *r*=-1.294, SE=0.151, *p*<.001 | *r*=-0.352, SE=0.211, *p*=.097 |
